# Development and validation of a model to predict rebleeding within three days after endoscopic hemostasis for high-risk peptic ulcer bleeding

**DOI:** 10.1186/s12876-022-02145-9

**Published:** 2022-02-14

**Authors:** Yongkang Lai, Yuling Xu, Zhenhua Zhu, Xiaolin Pan, Shunhua Long, Wangdi Liao, Bimin Li, Yin Zhu, Youxiang Chen, Xu Shu

**Affiliations:** 1 Department of Gastroenterology, The First Affiliated Hospital of Nanchang University, 17 Yongwaizheng Street, Nanchang, 330006 Jiangxi Province China; 2 First School of Clinical Medicine, Nanchang University, Nanchang, 330006 Jiangxi China

**Keywords:** Peptic ulcer bleeding, Emergency endoscopic hemostasis, Nomogram, LASSO, Rebleeding

## Abstract

**Background:**

Peptic ulcer bleeding remains a typical medical emergency with significant morbidity and mortality. Peptic ulcer rebleeding often occurs within three days after emergent endoscopic hemostasis. Our study aims to develop a nomogram to predict rebleeding within three days after emergent endoscopic hemostasis for high-risk peptic ulcer bleeding.

**Methods:**

We retrospectively reviewed the data of 386 patients with bleeding ulcers and high-risk stigmata who underwent emergent endoscopic hemostasis between March 2014 and October 2018. The least absolute shrinkage and selection operator method was used to identify predictors. The model was displayed as a nomogram. Internal validation was carried out using bootstrapping. The model was evaluated using the calibration plot, decision-curve analyses, and clinical impact curve.

**Results:**

Overall, 386 patients meeting the inclusion criteria were enrolled, with 48 patients developed rebleeding within three days after initial endoscopic hemostasis. Predictors contained in the nomogram included albumin, prothrombin time, shock, haematemesis/melena and Forrest classification. The model showed good discrimination and good calibration with a C-index of 0.854 (C-index: 0.830 via bootstrapping validation). Decision-curve analyses and clinical impact curve also demonstrated that it was clinically valuable.

**Conclusion:**

This study presents a nomogram that incorporates clinical, laboratory, and endoscopic features, effectively predicting rebleeding within three days after emergent endoscopic hemostasis and identifying high-risk rebleeding patients with peptic ulcer bleeding.

*Trial registration* This clinical trial has been registered in the ClinicalTrials.gov (ID: NCT04895904) approved by the International Committee of Medical Journal Editors (ICMJE).

**Supplementary Information:**

The online version contains supplementary material available at 10.1186/s12876-022-02145-9.

## Introduction

Peptic ulcer bleeding (PUB) is the major cause of acute non-variceal upper gastrointestinal bleeding (NVUGIB), which remains an urgent medical problem with significant morbidity and mortality [1–4]. Although the prognosis of patients with PUB has improved with advances in proton pump inhibitors (PPIs) therapy and endoscopic treatment, it remains a potentially life-threatening gastrointestinal emergency. Many patients died every year due to peptic ulcer rebleeding [5–7]. Through our clinical observation, we found that most of the rebleeding occurred within three days after endoscopic hemostasis, and the death basically occurred in the people rebleeding within three days (see Additional file 1). Therefore, exploring efficient factors and developing tools for early identifying patients with a high risk of rebleeding after emergent endoscopic hemostasis is an adequate precaution to improve the prognosis of PUB.

Previous studies developed several scoring systems to estimate the prognosis of patients with upper gastrointestinal bleeding (UGIB), including the Glasgow-Blatchford score (GBS), Rockall score (RS), and the AIMS65 score [8–10]. However, the RS and the AIMS65 score were mainly developed to estimate the mortality risk of patients [11, 12]. As for the GBS, by summarizing much research, the guideline suggested using GBS ≤ 1 to identify patients at low risk for rebleeding or mortality [4, 5, 13]. Besides, the predictive ability of GBS in patients with high-risk ulcers after endoscopic hemostasis is unsatisfactory [11, 13–15]. In addition, these scoring systems’ complexity has limited their application in routine clinical situations.

Prior researches have been carried out to determine predictors of rebleeding in patients with PUB, and some have been incorporated into predictive models. Factors found to be predictors of rebleeding include Forrest classification, use of omeprazole, liver cirrhosis, recent surgery, systolic blood pressure below 100 mmHg, heart rate above 100 bpm, hematemesis, large ulcer size and ulcer site [16–20]. However, study predicting rebleeding within three days after emergency endoscopic hemostasis is rare. Besides, the predictive ability of models mentioned above was unsatisfactory. Thus, the study aimed to establish a novel prediction model displayed as a nomogram to predict rebleeding after emergency endoscopic hemostasis for PUB.

## Methods

### Patients and study design

This was a retrospective study. Patients who underwent emergent endoscopic for peptic ulcers with high-risk stigmata (Forrest Ia–Forrest IIb. Patients with more than one ulcer were classified as the most severe ulcer.) and performed endoscopic hemostasis at the Department of Gastroenterology, the First Affiliated Hospital of Nanchang University between March 2014 and October 2018 were enrolled. The exclusion criteria for this study were as follows: (1) Patients with Forrest IIc and III peptic ulcers, which did not require endoscopic therapy; (2) demographic data was incompleted. Then we collected patients’ information, including demographic information, physical examinations, clinical characteristics, auxiliary examination findings, the GBS, the RS, the AMIS65 score and clinical outcomes. The study protocol was approved by the review boards of The First Affiliated Hospital of Nanchang University center (No: 2021058).

The study outcome was rebleeding within three days of the initially successful therapeutic endoscopy. Rebleeding was defined as recurrent hematemesis or melena with a decrease in hemoglobin by at least 2 g/dL within three days after the initial endoscopic treatment [19], and all patients with rebleeding were confirmed with a second look of endoscopy. Shock was defined as shock index (pulse rate/systolic blood pressure) > 1.0. We classified patients with only hematemesis or both hematemesis and melena as hematemesis group, and classified patients with melena as melena group.

### Endoscopic evaluation and pharmacologic therapy

Experienced endoscopists performed all emergent endoscopies within 12 h of hospital admission. A single-channel endoscope (GIF-XQ290, Olympus Optical Co., Ltd., Japan) was used during the procedure. The endoscopic hemostasis methods included injection therapy, thermal coagulation, mechanical therapy and combined therapy. After successfully hemostasis, the patients would receive high-dose intravenous proton pump inhibitors (80 mg of intravenous injection, then continuous infusion of 8 mg per hour for 72 h.). Then, the patients would receive 40 mg esomeprazole once daily for 30 days.

### Statistical analysis

For normally distributed data, continuous variables were presented as the mean ± standard deviation (SD) and the differences between the rebleeding and no-rebleeding groups were compared using Student’s t-test. For non-normal distributed data, continuous variables were presented as the median and interquartile range (IQR) and the Mann–Whitney rank-sum test was used to analyze the difference between the two groups. Categorical variables were presented as proportions, and the chi-square test or Fisher’s exact test was used accordingly.

The least absolute shrinkage and selection operator (LASSO) method, which is suitable for the regression of high-dimensional data [21], was used to select the most useful predictive features from the primary data set. And we used the 1 standard error of the minimum criteria (the 1-SE criteria) value as cutoff. Albumin, prothrombin time (PT), shock, haematemesis/melena and Forrest classification were used to construct a nomogram.

Next, calibration curves and the concordance index (C-index) were calculated to evaluate the performance of the model in predicting prognosis. The values of C index of 0.5 and 1.0 respectively represent the random chance and good ability of the model to predict rebleeding. Besides, decision-curve analysis (DCA) and clinical impact curve were also used to determine the clinical net benefit associated with the use of the model [22]. Finally, the model was internally validated via bootstrapping resampling of the construction data set (with 1000 bootstrap samples per model) to obtain optimism corrected discrimination via the C-index for rebleeding [23]. What’s more, we also compared the Area Under Curve (AUC) for the models vs. three clinical risk scores (GBS, RS, and the AIMS65). *P* < 0.05 was considered to be statistically significant. All the statistical analyses were performed by R statistical software 4.1.0 (www.r-project.org).

## Results

### Clinical characteristics

**Fig. 1:**
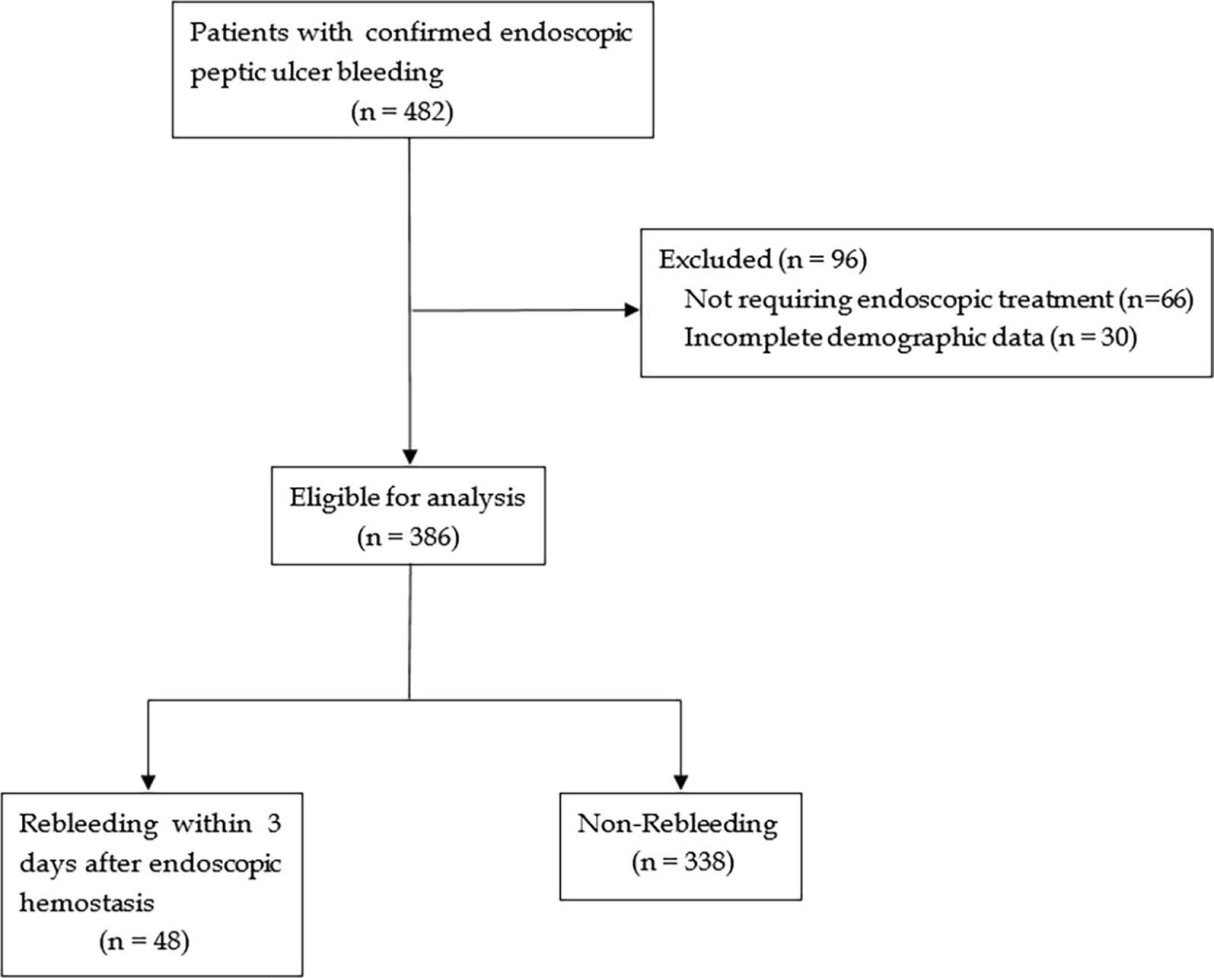
The flowchart of patients included in the present study

A total of 386 patients with PUB who underwent emergency endoscopic hemostasis during the study period at our center were enrolled (Fig. 1). Among these patients, 48 had rebleeding within three days after initial endoscopic hemostasis. The included patients’ median age (IQR) was 56 (43–65) years old, and 313 (81.1%) of these patients were male. The enrolled patients’ baseline characteristics are shown in Tables 1, 2 and 3. Compared to patients who did not rebleed, patients who rebled were more likely to present haematemesis and shock at the time of admission. What is more, patients in the rebleeding group seemed to have a faster heart rate, higher AIMS65 score, higher white cell count, lower platelet, lower albumin, prolonged PT, prolonged activated partial thromboplastin time (APPT) and international normalized ratio (INR) (all *P* < 0.05). While no differences were observed between the rebleeding group and non-rebleeding group concerning age, sex, alcohol use, smoking, medication use, PU bleeding history, hypertension, diabetes mellitus, systolic blood pressure, diastolic blood pressure, GBS, Rockall score, haemoglobin level on admission, blood urea nitrogen, creatinine, ulcer location, ulcer size ≥ 2 cm and methods of endoscopic hemostasis.

**Table 1:** Overall Baseline Characteristics and Comparison between rebleeding and no-rebleeding group

Characteristic	Total	Rebleeding	Non-rebleeding	*P*
	N = 386	N = 48	N = 338	
Age, median (IQR)	56 (43–65)	54 (31.5–65)	56 (43–65)	0.229
Sex: male [No. (%)]	313 (81.1)	37 (77.1)	276 (81.7)	0.555
Alcohol use [No. (%)]	64 (16.6)	5 (10.4)	59 (17.5)	0.299
Smokers [No. (%)]	119 (30.8)	9 (18.8)	110 (32.5)	0.077
Haematemesis [No. (%)]	194 (50.3)	40 (83.3)	154 (45.6)	0.001
Medication history				
Use of NSAIDs [No. (%)]	22 (5.7)	2 (4.2)	20 (5.9)	0.875
Use of anticoagulants [No. (%)]	4 (1)	0	4 (1.2)	1
PU bleeding history [No. (%)]	73 (18.9)	5 (10.4)	68 (20.1)	0.108
Coexisting diseases [No. (%)]				
Hypertension	90 (23.3)	10 (20.8)	80 (23.7)	0.664
Diabetes mellitus	34 (8.8)	3 (6.3)	31 (9.2)	0.692
Shock [No. (%)]	53 (13.7)	23 (47.9)	30 (8.9)	< 0.001
Systolic blood pressure [mmHg, median (IQR)]	114 (103–128)	110 (96–131.5)	114 (104–127)	0.484
Diastolic blood pressure (mmHg, mean ± SD)	70.14 ± 0.68	68.88 ± 2.37	70.32 ± 0.70	0.560
Heart rate [beats/min, median (IQR)]	86 (73–99)	99 (80.5–109.5)	85 (73–97)	< 0.001
Surgery due to rebleeding [No. (%)]	15 (3.9)	15 (31.1)	0	< 0.001
Mortality [No. (%)]	16 (4.1)	16 (33.3)	0	< 0.001
Blood transfusion [No. (%)]	192 (49.7)	33 (68.8)	159 (47)	0.005
Hospitalization stay, median (IQR)	7 (5–10)	12 (7–20)	6 (5–9)	< 0.001
Glasgow-Blatchford score, median (IQR)	10 (8–12)	10 (8–13)	10 (8–12)	0.138
Rockall score, median (IQR)	4 (3–4)	4 (3–5)	4 (3–4)	0.074
AIMS65 score, median (IQR)	1 (0–1)	2 (1–3)	0 (− 1)	< 0.001

**Table 2:** Laboratory findings and comparison between rebleeding and no-rebleeding group

Characteristic	Total	Rebleeding	Non-rebleeding	*P*
	N = 386	N = 48	N = 338	
Hemoglobin level on admission [g/L, median (IQR)]	78.5 (64–98)	74.5 (59.5–93)	79 (66–98)	0.124
White cell count [× 109/L, median (IQR)]	7.85 (5.65–11..11)	9.35 (7.12–12.81)	7.59 (5.65–10.92)	0.019
Platelet [× 109/L, median (IQR)]	167 (116–218)	132.5 (89.5–192)	172.5 (121–219)	0.015
Blood urea nitrogen [mmol/L, median (IQR)]	8.3 (5.7–12.3)	10.25 (5.65–15.15)	8.2 (5.7–11.94)	0.092
Creatinine [μmol/L, median (IQR)]	74.2 (61.3–90.8)	72.6 (59.9–107.6)	74.9 (61.3–89.3)	0.596
Albumin [g/L, median (IQR)]	32 (27.9–37)	28.8 (24.9–32)	32.4 (28.8–37.2)	0.001
Albumin [ALB ≤ 30 g/L, No. (%)]	124 (32.1)	25 (52.1)	99 (29.3)	0.002
Prothrombin time [s, median (IQR)]	11.8 (11–13)	13.1 (11.6–16.2)	11.7 (11–12.8)	0.001
APTT, median (IQR)	28.65 (24.5–33.1)	32.15 (27.15–50.5)	28.35 (24.5–32.5)	0.001
International normalized ratio [INR > 1.5, No. (%)]	15 (3.9)	8 (16.7)	7 (2.1)	0.001

**Table 3:** Endoscopic findings and comparison between rebleeding and no-rebleeding group

Characteristic	Total	Rebleeding	Non-rebleeding	*P*
	N = 386	N = 48	N = 338	
Ulcer location [No. (%)]				0.282
Fundus	18 (4.7)	1 (2.1)	17 (5)	
Body	63 (16.3)	7 (14.6)	56 (16.6)	
Angulus	18 (4.7)	1 (2.1)	17 (5)	
Antrum	41 (10.6)	4 (8.3)	37 (10.9)	
Duodenum	193 (50)	32 (66.7)	161 (47.6)	
Anastomotic site	53 (13.7)	3 (6.3)	50 (14.8)	
Ulcer size ≥ 2 cm [No. (%)]	35 (9.1)	7 (14.6)	28 (8.3)	0.249
Stigmata of hemorrhage [No. (%)]				0.001
Forrest Ia	22 (5.7)	7 (14.6)	15 (4.4)	
Forrest Ib	144 (37.3)	26 (54.2)	118 (34.9)	
Forrest IIa	121 (31.1)	7 (14.6)	114 (33.7)	
Forrest IIb	99 (25.6)	8 (16.7)	91 (29.6)	
Methods of endoscopic hemostasis [No. (%)]				0.453
Injection therapy	224 (58)	30 (62.5)	194 (57.4)	
Thermal coagulation	18 (4.7)	2 (4.2)	16 (4.7)	
Mechanical therapy	60 (15.5)	4 (8.3)	56 (16.6)	
Combination therapy	84 (21.8)	12 (25)	72 (21.3)	

### Feature selection based on LASSO method

**Fig. 2:**
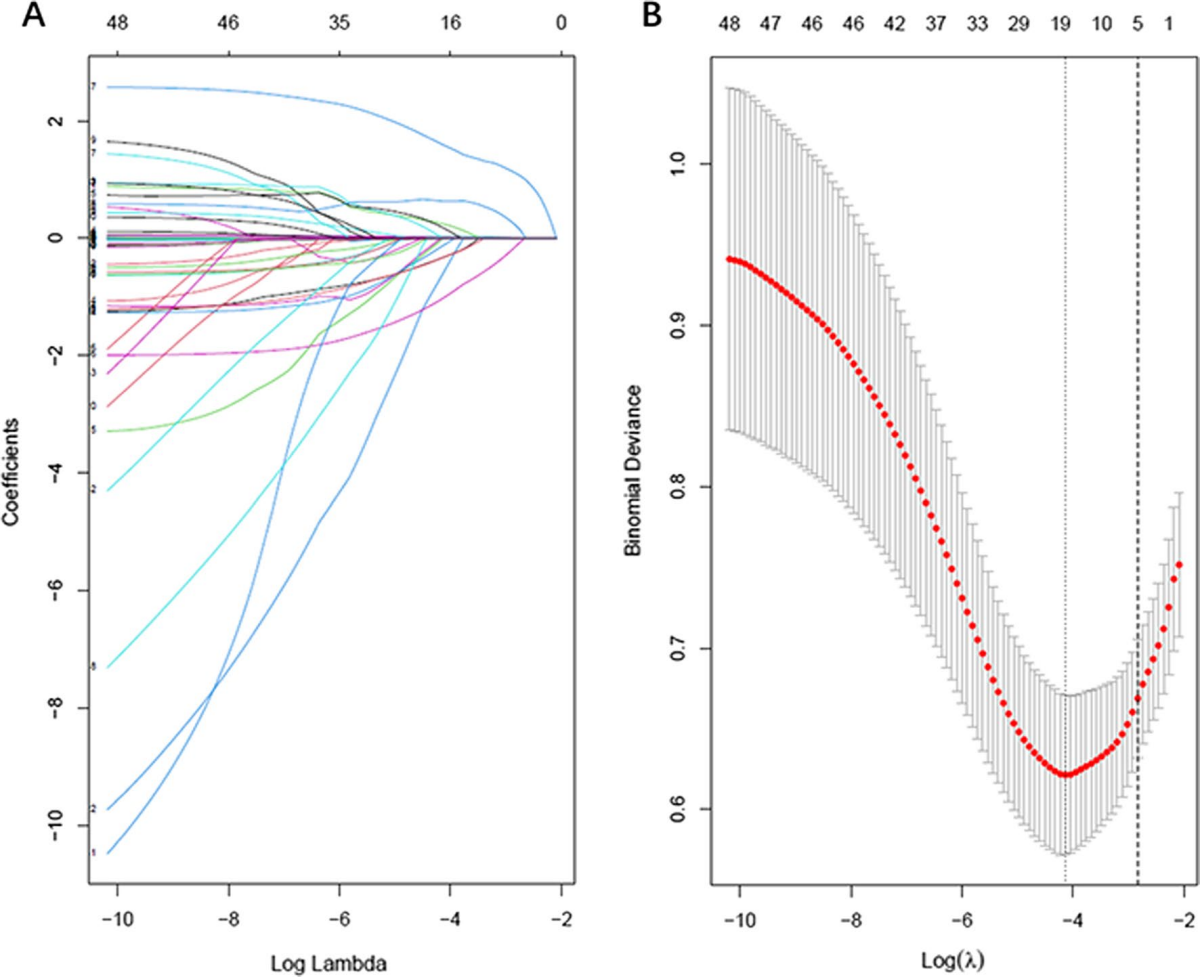
Predictors selection based on the least absolute shrinkage and selection operator (LASSO) regression. **A** LASSO Select Model ([lambda]) of the adjustment parameter by the minimum standard, and tenfold cross-validation. Use log(λ) to plot the area under the receiver operating characteristic curve. Draw a vertical dashed line at the optimal value with one standard error of the minimum standard and 1 standard error of the minimum standard (1-SE standard). **B** The tuning parameter (lambda) selection in LASSO regression uses tenfold cross-validation. The binomial deviation is plotted on the logarithm (lambda). Use the 1-SE standard to draw a dashed line at the optimal value

Rebleeding within three days after emergency endoscopic hemostasis was chosen as the study outcome. In order to reduce the dimensionality and screen out the most representative risk factors for PU rebleeding within three days after endoscopic hemostasis, LASSO regression analysis was performed on the 48 collected variables using the 1-SE criteria value as the cutoff. And as a result, five variables that predict rebleeding within three days after the initially successful therapeutic endoscopy for PU screened out, including albumin, PT, shock, haematemesis/melena, Forrest classification (Fig. 2A, B).

### Development and assessment of the nomogram

**Fig. 3:**
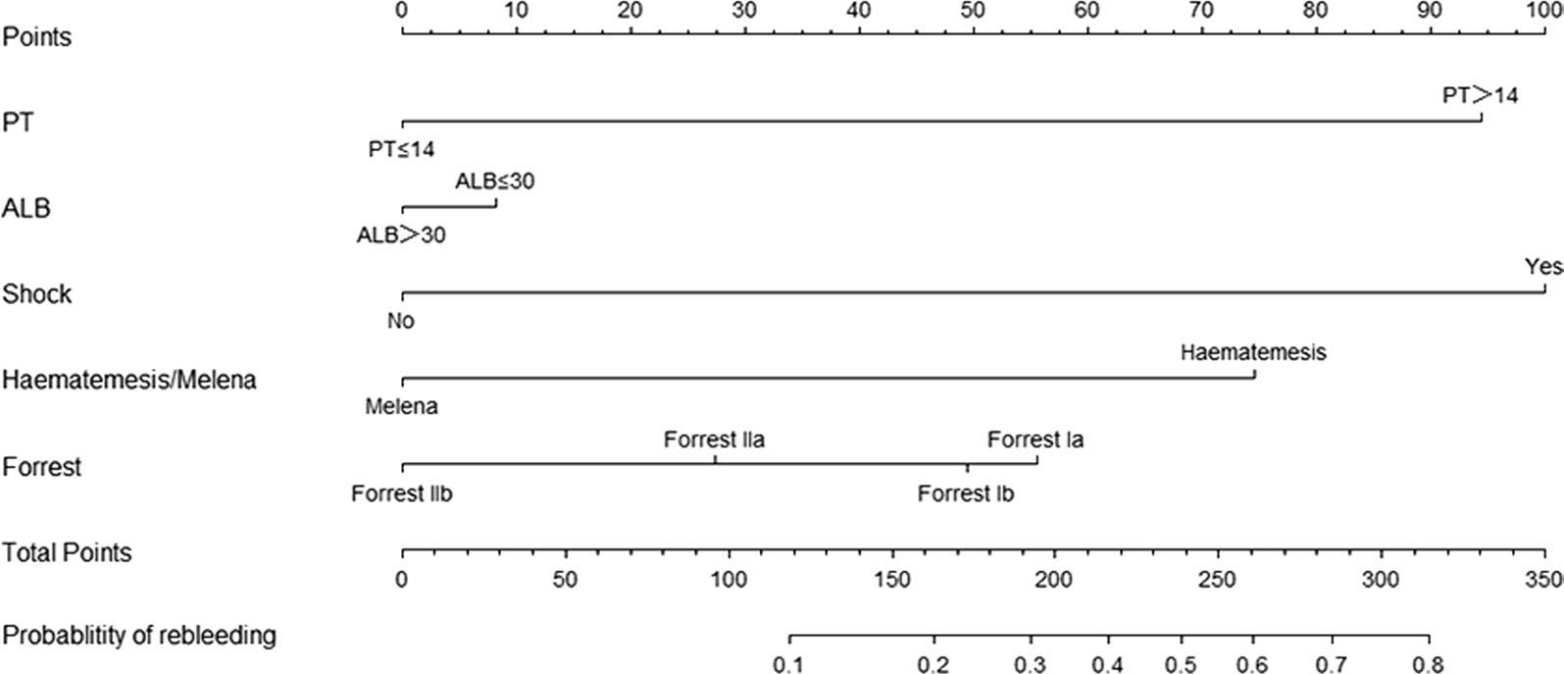
Nomogram predicting the probability of rebleeding within three days following endoscopic therapy for peptic ulcer bleeding

To predict rebleeding within three days after emergency endoscopic hemostasis for PUB, we conducted multivariable logistic regression analysis using the five predictors selected by the LASSO method. And construct an accurate and stable nomogram (Fig. 3). The equation built for model was LogitP = − 0.148–0.21‬ * albumin (ALB ≤ 30 = 1) + 1.763 * PT (PT ≥ 14 = 1) + 1.873 * shock (shock = 1) − 1.363 * haematemesis/melena (haematemesis = 1/melena = 2) − 0.281 * Forrest classification (Forrest Ia = 1, Forrest Ib = 2, Forrest Iia = 3, and Forrest IIb = 4). (Table 4).

**Table 4:** Multivariate regression analysis

Variables	B	S.E.	OR	Lower 95% CI	Higher 95% CI	*P* value
Albumin	− 0.21	0.4	0.811	0.371	1.774	0.6
PT	1.763	0.43	5.828	2.507	13.548	0.001
Shock	1.873	0.402	6.508	2.959	14.318	0.001
Haematemesis/Melena	− 1.363	0.437	0.256	0.109	0.602	0.002
Forrest classification	− 0.281	0.205	0.755	0.506	1.128	0.171
C-index						
Primary cohort					0.854	
Internal validation (with 1000 bootstrap samples per model)					0.830	

**Fig. 4:**
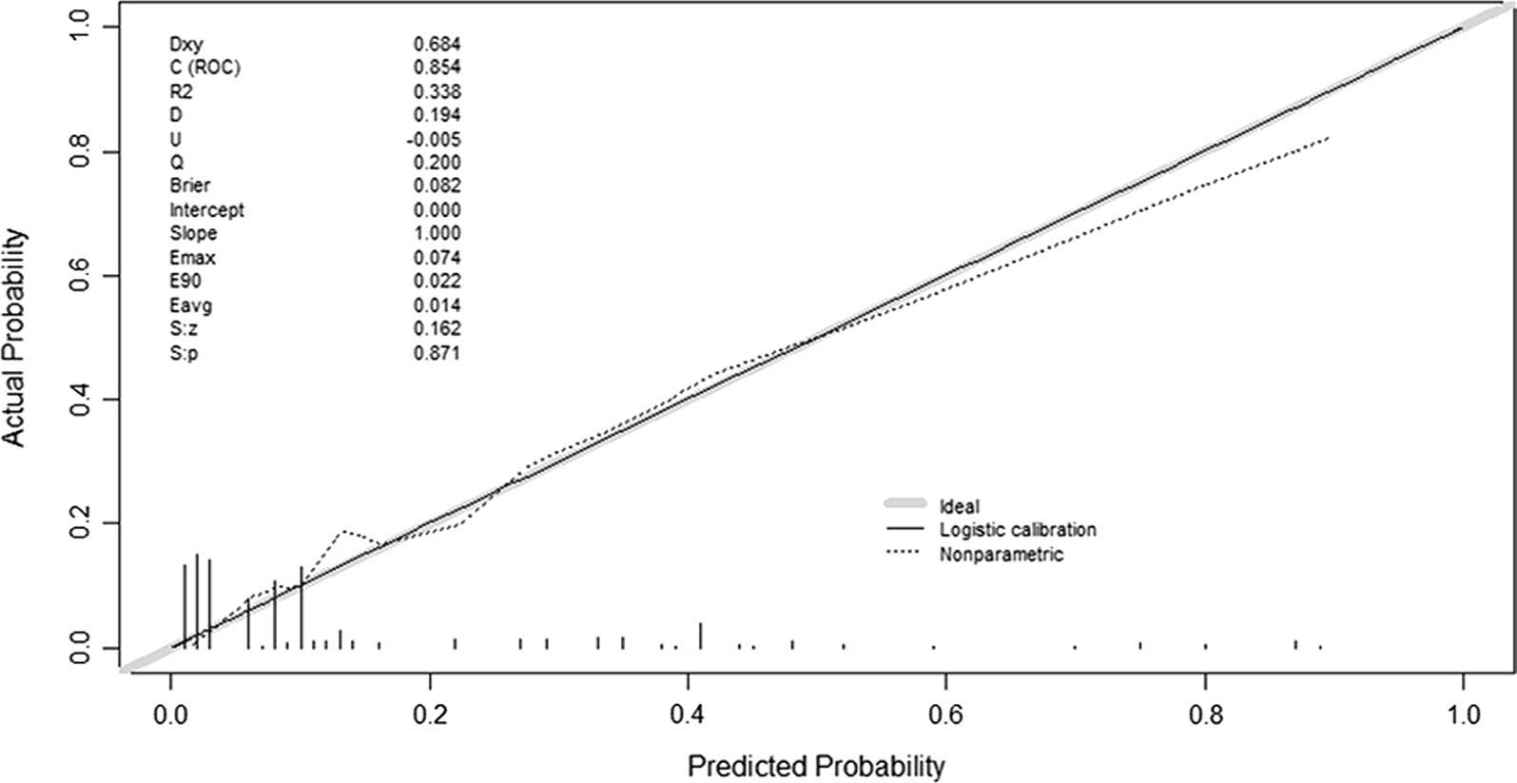
Calibration curves of the nomogram in the primary cohort

The calibration curve of the predictive model showed a good fit between the prediction and observation in the primary cohort (Fig. 4). The Hosmer–Lemeshow test yielded a nonsignificant statistic (*P* = 0.716), showing that the model worked well. The C-index for the predictive model was 0.854, which suggested the model had a good predictive ability.

**Fig. 5:**
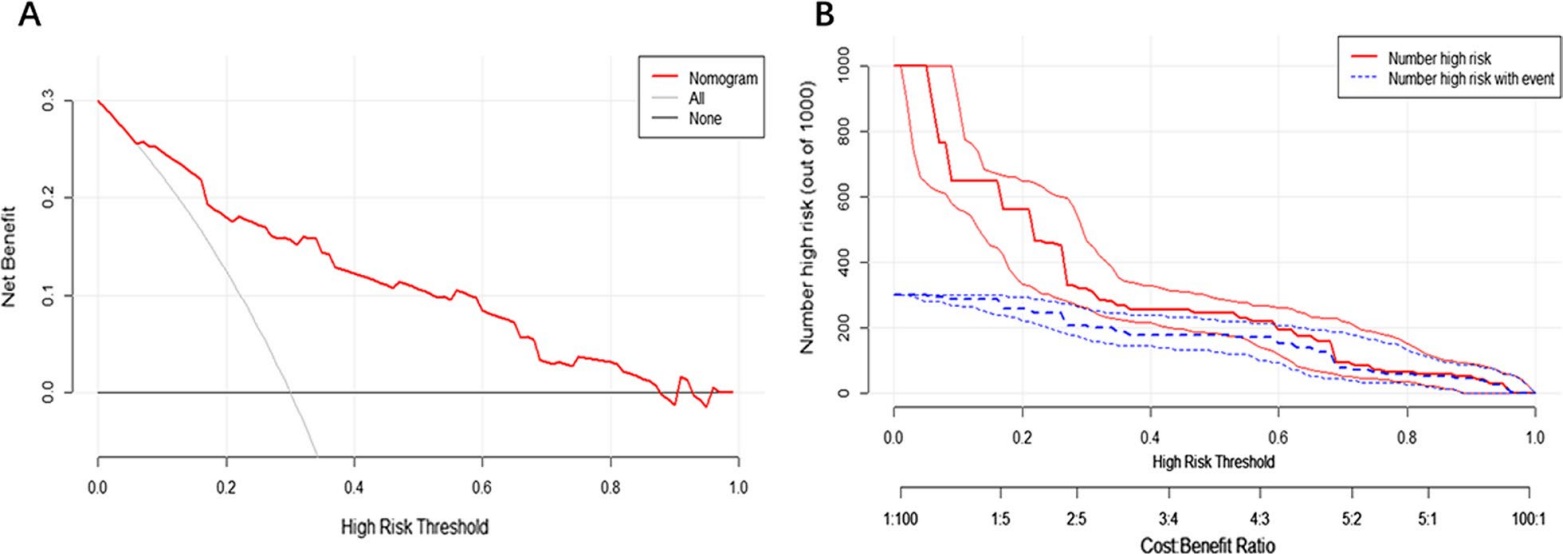
**A** Decision curve analysis for the nomogram; **B** clinical impact curve for the nomogram

**Fig. 6:**
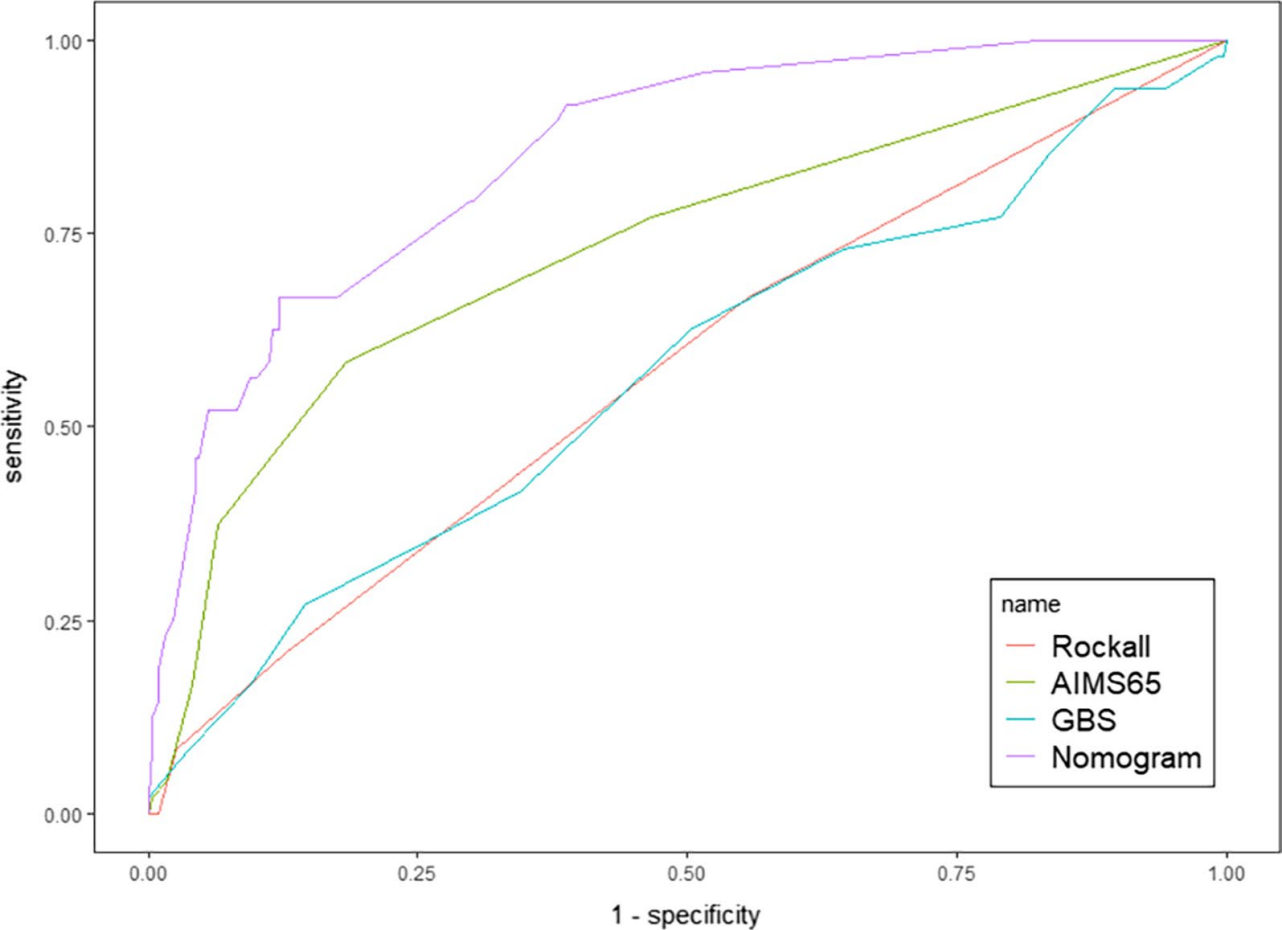
Comparison of ROC among the nomogram, Glasgow-Blatchford score system, Rockall score system and AIMS65 score system. *ROC* receiver operating characteristic, *GBS* Glasgow-Blatchford score

The DCA demonstrated that this model improved patient outcomes compared with either treat-all or treat-none strategies by helping assess the risk of rebleeding in patients and informing interventions (Fig. 5A). The DCA showed more benefit in the current study with a threshold probability > 0.0% using the nomogram. Besides, the clinical impact curve for the model was also visually indicated that nomogram conferred high clinical net benefit and confirmed the clinical value of this model (Fig. 5B). And the model also performed better than GBS, RS, and AIMS65 (Fig. 6).

### Internal validation

Finally, this model was internally validated using bootstrapping resampling of the construction data set (with 1000 bootstrap samples per model). Moreover, the C-index for this nomogram was 0.830, which suggested high accuracy.

## Discussion

PUB is the most common cause of acute non-variceal upper gastrointestinal bleeding. Although the development of endoscopic technology has effectively improved the prognosis of PUB, rebleeding is still one of the common complications [4]. Therefore, it is imperative for clinicians to identify high-risk rebleeding patients after emergent endoscopic therapy, for which clinicians can give more powerful measures. According to our clinical observation, high-risk peptic ulcer rebleeding often occurs within three days after emergent endoscopic hemostasis. However, to our knowledge, there were few studies focused on rebleeding within three days. Anne C Travis et al. developed a model to predict rebleeding for NVUGIB. However, this model's study outcome was rebleeding within 30 days of the initially successful therapeutic endoscopy, and the predictive ability of this model was barely satisfactory (AUC = 0.752) [24]. Zhiyu Dong et al. established a new scoring system to predict poor clinical outcomes for NVUGIB, while this study had a small sample size and the predictive ability is still low [25]. There were also other clinical trials carried out to build models to predict rebleeding for PUB. However, these studies either did not focused on rebleeding within three days after emergent endoscopic hemostasis or had the poor predictive ability [18, 26–28]. Hence, in the present study, we developed a novel model to predict rebleeding within three days after emergent endoscopic hemostasis for high-risk peptic ulcer bleeding, and the model showed an excellent discriminatory ability (C-index: 0.854).

In the present study, five predictors identified by the LASSO method were incorporated into the nomogram, and the model was proven to be of excellent performance in internal validation. The Forrest classification is mainly used to stratify ulcer bleeding patients and guide management decisions, including endoscopic and pharmacological therapy. Moreover, many studies proved that the Forrest classification had excellent predictive value for rebleeding peptic ulcers [4, 18, 26]. Thus the Forrest classification is a stronger predictor for rebleeding in PUB. Hypoalbuminemia is a risk factor of mortality in certain diseases, and the correlation between hypoalbuminemia and the prognosis of PUB has been reported. Hsiu-Chi Cheng et al. indicated that hypoalbuminemia in patients with peptic ulcer bleeding could be an alarm indicator of recurrent bleeding [29]. In our study, albumin ≤ 30 g/L scored more in the nomogram than albumin > 30 g/L, which is similar to the previous study. Patients admitted to the hospital with hematemesis and shock heralded more dangerous gastrointestinal bleeding and heralded a worse prognosis [4]. Hence, hematemesis and shock scored more in the nomogram. Prolonged PT indicates deranged coagulation function and was another valuable predictor for rebleeding in patients presenting with PUB [30]. For patients with prolonged PT, the doctor should pay more attention to or take an extra intervention.

There were several advantages in the present study. First, this is the first study to construct a model that incorporated variables from clinical, laboratory, endoscopic features for predicting rebleeding within three days after initial endoscopic therapy for high-risk PUB. Second, informative variables were identified using the LASSO method, which can avoid the statistical defects of overfitting compared with using univariate analysis. Third, our model had an excellent predictive ability. What is more, the model was displayed as a nomogram which was intuitive and easy to use in clinical practice.

However, there were some limitations in the present study. First, the present study was a single-centre retrospective study. Second, our study only had internal validation. However, the model had a good performance in predictive ability (C-index: 0.854), and the internal verification performance was also good (C-index via bootstrapping validation: 0.830). This model needs to be prospectively validated on a distinct group of patients in the future.

## Conclusion

In conclusion, we established and internally validated a nomogram to predict rebleeding within three days after emergent endoscopic hemostasis. This nomogram incorporated variables from clinical, laboratory and endoscopic features and can be conveniently used to identify high-risk patients after emergent endoscopic hemostasis, which can help doctors pay more attention to or give extra intervention.

## Supplementary Information

**Additional file 1:** Patients mortality distribution.

## Data Availability

The datasets used and/or analysed during the current study are available from the corresponding author on reasonable request.

## Abbreviations

ICMJE: The International Committee of Medical Journal Editors
PUB: Peptic ulcer bleeding
NVUGIB: Non-variceal upper gastrointestinal bleeding
PPIs: Proton pump inhibitors
GBS: Glasgow-Blatchford score
RS: Rockall score
UGIB: Upper gastrointestinal bleeding
IQR: Interquartile range
SD: Standard deviation
LASSO: The least absolute shrinkage and selection operator
1-SE: 1 Standard error
PT: Prothrombin time
C-index: Concordance index
DCA: Decision-curve analysis
AUC: Area under curve
INR: International normalized ratio
APPT: Prolonged activated partial thromboplastin time
CI: Confidence interval
OR: Odds ratio
S.E.: Standard error
