# Supplementary material for: Development and validation of a model to predict rebleeding within three days after endoscopic hemostasis for high-risk peptic ulcer bleeding
Source: BMC Gastroenterol. 2022 Feb 14;22:64. doi: 10.1186/s12876-022-02145-9 (PMC8843020; doi:10.1186/s12876-022-02145-9)
Supplement: Supplementary file 1 — Additional file 1: Patients mortality distribution. [file 12876_2022_2145_MOESM1_ESM.docx]

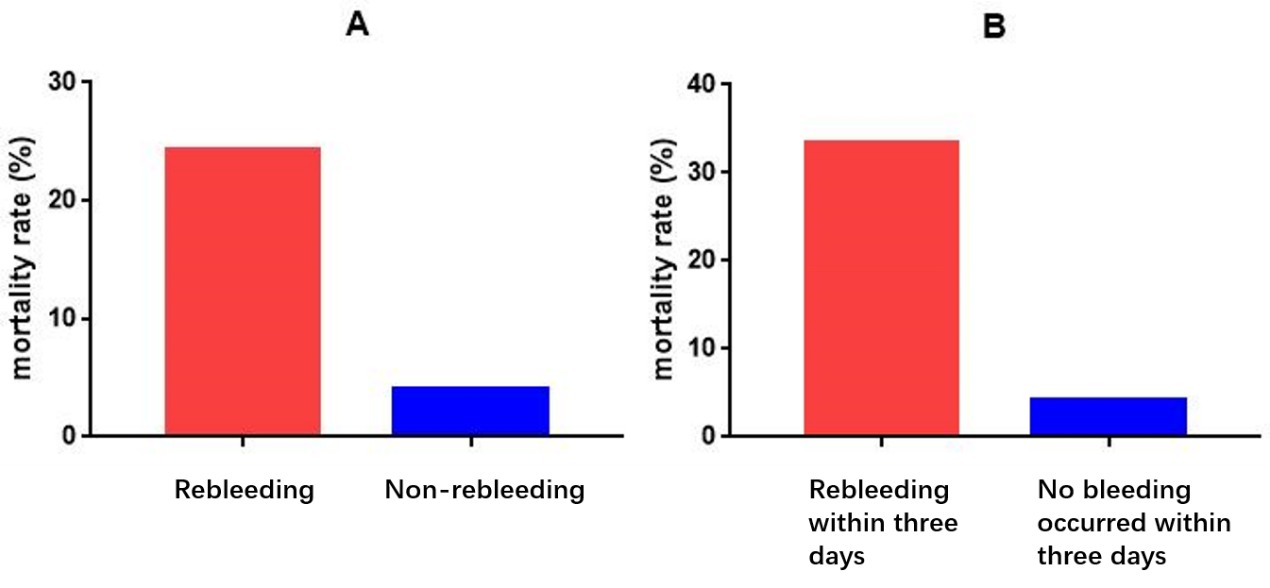


**Additional file 1** Patients mortality distribution. (A): Comparison of rebleeding related and non-rebleeding related mortality rate within 30 days after endoscopic hemostasis. (B): Comparison of rebleeding related mortality within 3 days after endoscopic hemostasis and non 3-day rebleeding related mortality after endoscopic hemostasis.
